# Supplementary material for: Improving the quality of maternal and newborn healthcare at the district level: Addressing newborn deaths in Nepal
Source: PLOS Glob Public Health. 2023 Aug 2;3(8):e0002101. doi: 10.1371/journal.pgph.0002101 (PMC10395984; doi:10.1371/journal.pgph.0002101)
Supplement: S1 Text — (DOCX) [file pgph.0002101.s003.docx]

**Additional file S1**

**Supplementary Information**

**Contents**

**Fig A.** Data linkage flow chart

**Fig B.** Conceptual framework

**Table A.** The definition of selected quality indicators

**Table B.** Collinearity check using VIF

**Table C.** Comparison of quality scores calculated from an additive indicators method and a PCA method

**Fig C.** Mean scores of 16 quality indicators

**Table D.** Association between the quality scores and newborn/perinatal deaths using median quality score

**Fig A.** Data linkage flow chart


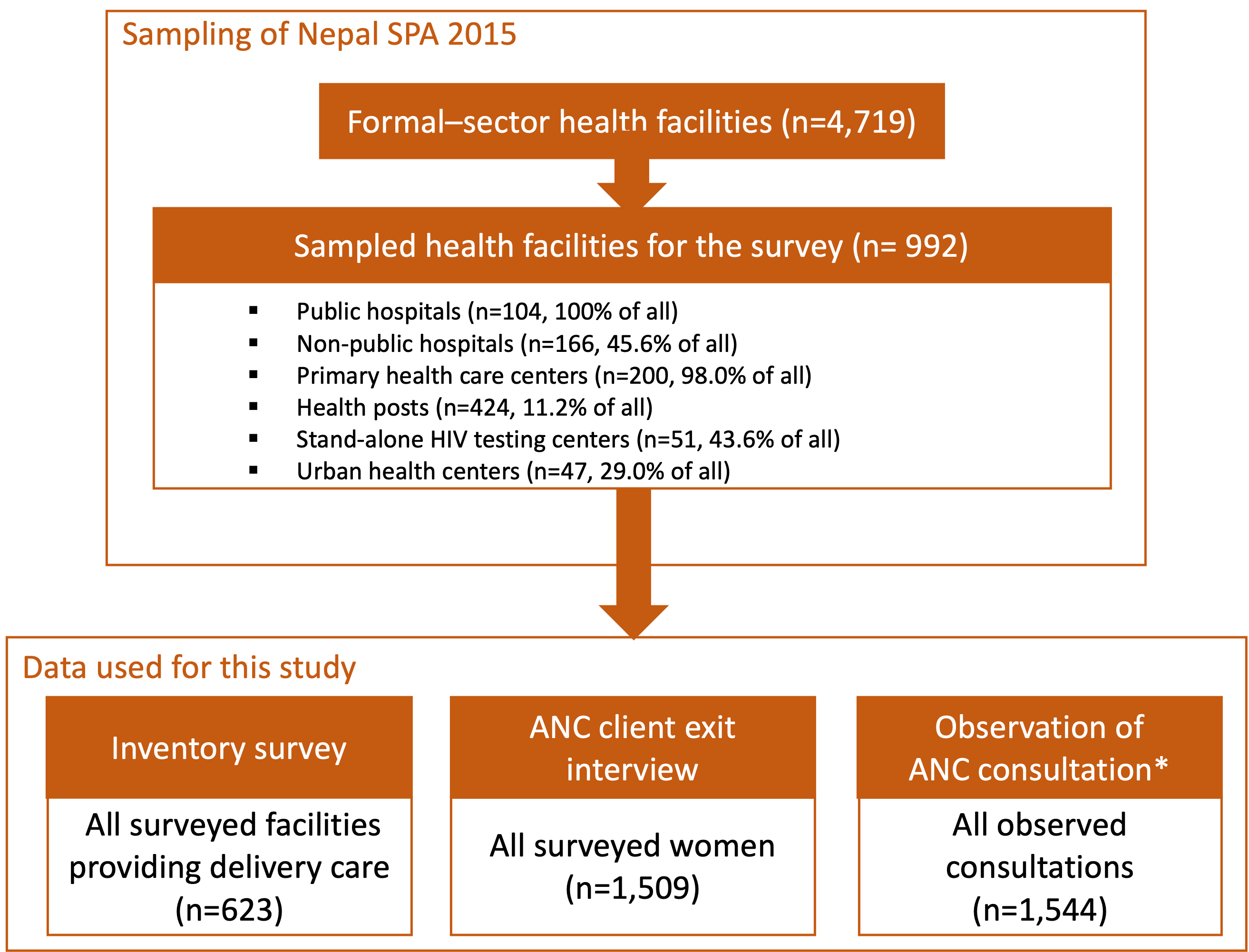


**Fig B.** Conceptual framework


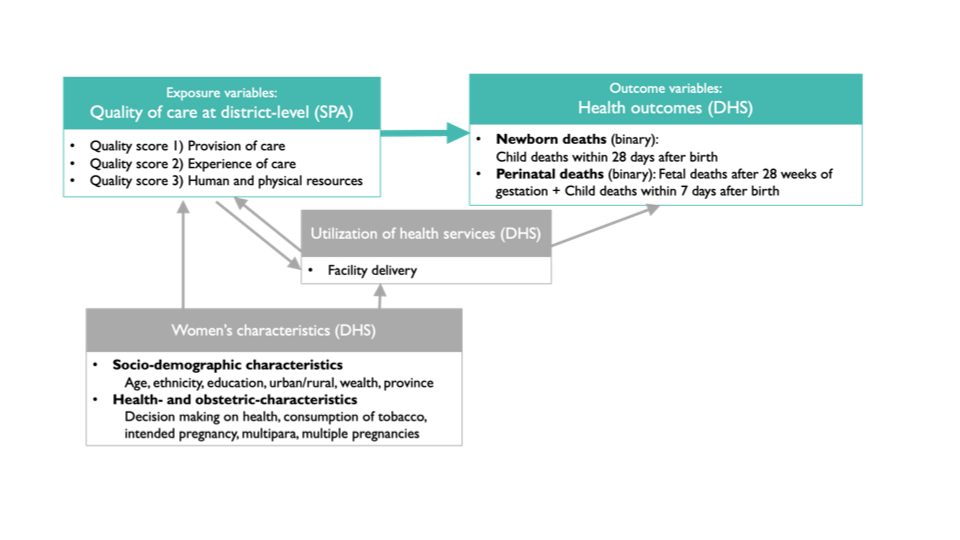


**Table A.**  **The definition of selected quality indicators**

| Dimension 1: Provision of care (n = 623) | | |  |  |  |  |  |
| --- | --- | --- | --- | --- | --- | --- | --- |
| Item | Indicator type | Indicator details | | | | | Missing values |
| 1-1. Partograph use | Binary | Blank partographs is observed | | AND | Provider answered that partograph is used routinely | | 0 |
| 1-2. Capacity to remove retained products | Binary | Removal of retained products were ever provided | | | | | 0 |
| 1-3. Parental oxytocin for hemorrhage in the last three months | Binary | Used parental oxytocic drugs for hemorrhage in the last three months | | AND | At least one valid injectable uterotonic (oxytocin) is observed | | 0 |
| 1-4. Parental magnesium sulfate for (pre-)eclampsia in the last three months | Binary | Used parental anti-convulsant for hypertension in the last three months | | AND | At least one valid injectable magnesium sulfate is observed | | 0 |
| 1-5. Manual removal of placenta in the last three months | Binary | Used manual removal of placenta in the last three months | | | | | 0 |
| 1-6. Parental antibiotics for maternal infection in the last three months | Binary | Used parental antibiotics for maternal infection in the last three months | | AND | At least one valid injectable antibiotic is observed | | 0 |
| 1-7. Neonatal resuscitation in the last three months | Binary | Provided neonatal resuscitation in the last three months | | | | | 0 |
|  |  |  |  |  |  |  |  |
| Dimension 2: Experience of care (data from the ANC interview: n = 1,509, data from the ANC observation: n = 1,544) | | | | | | | |
| Item | Indicator type | Indicator details | | | | | Missing values |
| 2-1. Perception of women* | Continuous (mean of the indicators on the right) | No problem in i) waiting time, ii) ability to discuss problems/concerns, iii) amount of explanation you received about the problem or treatment, iv) visual and auditory privacy, v) availability of medicines, vi) hours and days of service, vii) cleanliness of the facility, viii) how staff treat the client, ix) cost of services or treatment, and x) would recommend to friends/other family member | | | | | 35 |
| 2-2. Satisfaction of women* | Continuous (mean of the score in the right) | very satisfied = 5, satisfied = 4, neither satisfied nor dissatisfied = 3, fairly dissatisfied = 2, very satisfied = 0 | | | | | 35 |
| 2-3. Provider encouraged clients’ questions** | Binary | Provider asked if the client has any questions/encouraged questions | | | | | 0 |
| 2-4. Provider used visual aids during consultation** | Binary | Provider used visual aid during consultant | | | | | 0 |
| * Data from ANC client exit interview | | ** Data from observation of ANC consultation | | | |  |  |
|  |  |  |  |  |  |  |  |
| Dimension 3: Human and physical resources (n = 623) | | |  |  |  |  |  |
| Item | Indicator type | Indicator details | | | | | Missing values |
| 3-1. Availability of skilled provider for 24 hours | Binary | 24-hour staff coverage | | AND | Schedule of 24-hour staffing was observed | | 0 |
| 3-2. Referral resources | Binary | Functional ambulance with fuel is observed | | OR | Any functional communication system (landline or cell phone) observed during all service time | | 0 |
| 3-3. Electricity | Binary | Facility connected to central supply electricity grid and electricity is always available | | OR | functional backup generator with fuel reported | | 0 |
| 3-4. Water | Binary | Facility have access to improved water source (piped, public tap, standpipe, tubewell, borehole, protected dugwell, protected spring or rainwater) | | AND | water on site or within 500m of facility | | 0 |
| 3-5. Infectious control | Continuous (mean of the binary indicators on the right) | Delivery room has running water and soap | Delivery room has sharp container | Delivery room has disposable latex gloves | | delivery room has disinfectant | 0 |

**Table B.** Collinearity check using VIF

| Variable | VIF |
| --- | --- |
| Quality Score 1 | 1.70 |
| Age |  |
| 25–29 | Reference |
| ≤ 19 | 1.39 |
| 20–24 | 1.49 |
| 30–34 | 1.27 |
| 35–45 | 1.24 |
| Ethnicity |  |
| Brahmin/Chhetri | Reference |
| Other terai caste | 2.23 |
| Dalit | 1.45 |
| Janajati/Newar | 1.68 |
| Muslim/other | 1.56 |
| Education |  |
| Primary | Reference |
| No education | 1.98 |
| Secondary | 2.04 |
| Higher | 1.96 |
| Residence |  |
| Rural |  |
| Urban | 1.16 |
| Wealth index |  |
| Poorest | Reference |
| Poorer | 1.73 |
| Middle | 1.93 |
| Richer | 1.93 |
| Richest | 2.02 |
| Intended pregnancy |  |
| Then | Reference |
| Later/no more | 1.04 |
| Multipara |  |
| Yes | Reference |
| No | 1.46 |
| Involvement in decision-making on health | |
| Yes | Reference |
| No | 1.09 |
| Multiple pregnancies |  |
| Yes | Reference |
| No | 1.01 |
| Tobacco |  |
| Yes | Reference |
| No | 1.12 |
| Province |  |
| Province 1: Koshi | Reference |
| Province 2: Madhesh | 2.88 |
| Province 3: Bagmati | 1.63 |
| Province 4: Gandaki | 1.60 |
| Province 5: Lumbini | 2.25 |
| Province 6: Karnali | 2.30 |
| Province 7: Sudurpashchim | 1.93 |
| Mean VIF | 1.68 |

**Table C.**  **Comparison of quality scores calculated from an additive indicators method and a PCA method**

| Correlation coefficient for Quality Score 1 (Provision of care) | | |
| --- | --- | --- |
| (n = 623) |  |  |
|  | Quality Score 1  (Additive indicators method) | Quality Score 1  (PCA method) |
| Quality Score 1  (Additive indicators method) | 1.00 | - |
| Quality Score 1  (PCA method) | 0.99 | 1.00 |
|  |  |  |
| Correlation coefficient for Quality Score 2 (Experience of care) | | |
| (n = 1,509) |  |  |
|  | Quality Score 2  (Additive indicators method) | Quality Score 2  (PCA method) |
| Quality Score 2  (Additive indicators method) | 1.00 | - |
| Quality Score 2  (PCA method) | 0.74 | 1.00 |
|  |  |  |
| Correlation coefficient for Quality Score 3 (Human and physical resources) | | |
| (n = 623) |  |  |
|  | Quality Score 3  (Additive indicators method) | Quality Score 3  (PCA method) |
| Quality Score 3  (Additive indicators method) | 1.00 | - |
| Quality Score 3  (PCA method) | 1.00 | 1.00 |

**Fig C.**  **Mean scores of 16 quality indicators**


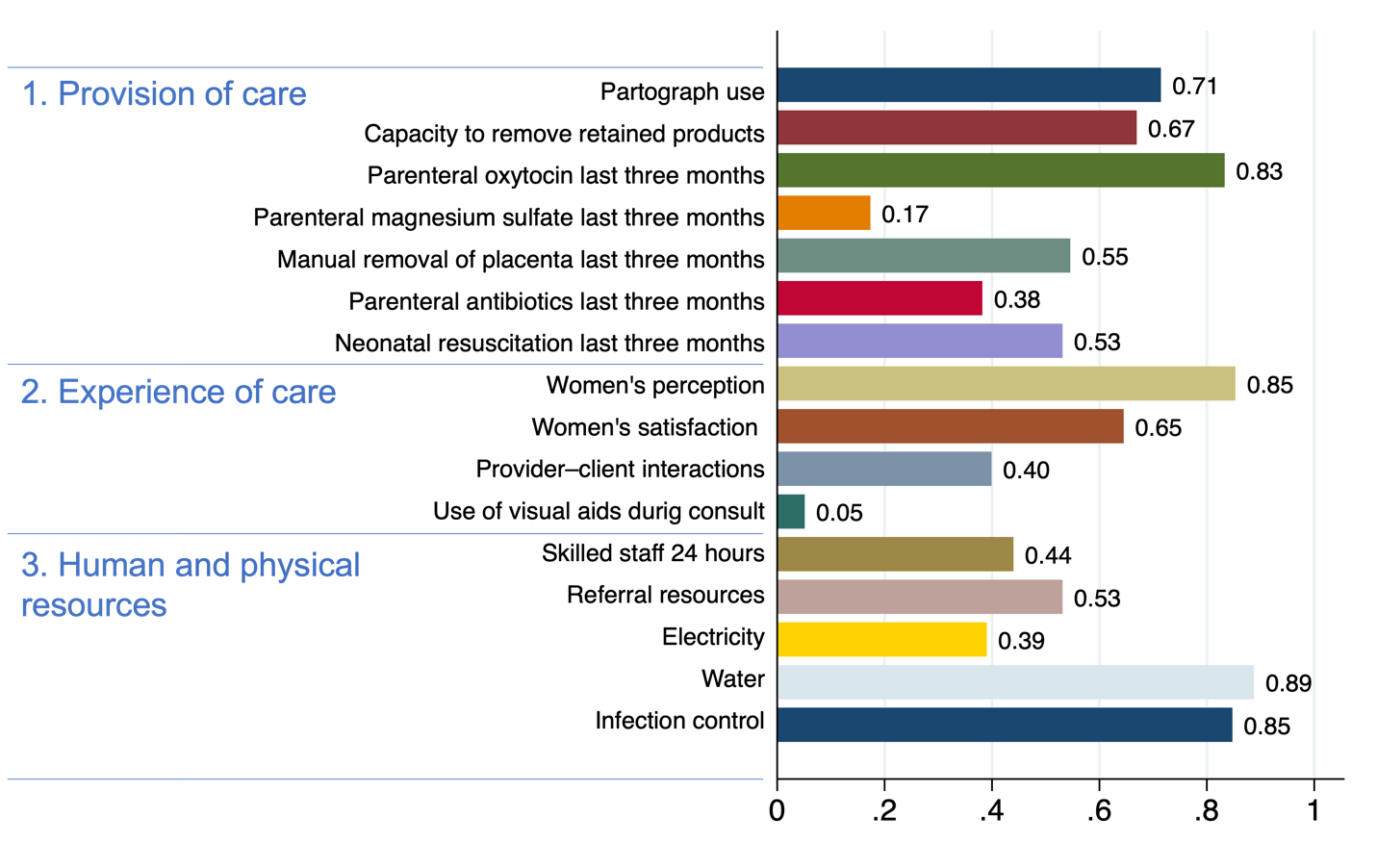


**Table D.**  **Association between the quality score and newborn/perinatal deaths using median quality score**

| **Quality dimension** | **Samples (weighted)** | **Newborn deaths** | | | | | | **Perinatal deaths** | | | | |
| --- | --- | --- | --- | --- | --- | --- | --- | --- | --- | --- | --- | --- |
|  |  | **Model 1** | |  | **Model 2*** | |  | **Model 1** | |  | **Model 2*** | |
|  |  | **OR**  **(95% CI)** | ***p*** |  | **AOR**  **(95% CI)** | ***p*** |  | **OR**  **(95% CI)** | ***p*** |  | **AOR**  **(95% CI)** | ***p*** |
| **Quality Score 1:**  **Provision of care** | **a. All cases** | 0.12 | **< .01** |  | 0.08 | **.02** |  | 0.43 | .26 |  | 0.42 | .35 |
|  | **(n = 5,060)** | (0.03–0.55) |  |  | (0.01–0.60) |  |  | (0.10–1.87) |  |  | (0.07–2.59) |  |
|  | **b. Facility delivery cases only** | 0.04 | **< .01** |  | 0.02 | **.02** |  | 0.93 | .95 |  | 1.58 | .78 |
|  | **(n = 2,157)** | (0.00–0.44) |  |  | (0.00–0.58) |  |  | (0.09–9.96) |  |  | (0.06–40.19) |  |
|  | **c. Non-facility delivery cases only** | 0.35 | .30 |  | 0.30 | .30 |  | 0.38 | .28 |  | 0.18 | .13 |
|  | **(n = 2,903)**** | (0.05–2.61) |  |  | (0.03–2.99) |  |  | (0.07–2.19) |  |  | (0.02–1.68) |  |
| **Quality Score 2: Experience of care** | **a. All cases** | 0.39 | .35 |  | 0.56 | .60 |  | 0.22 | .13 |  | 0.49 | .49 |
|  | **(n = 5,060)** | (0.05–2.87) |  |  | (0.06–4.96) |  |  | (0.03–1.57) |  |  | (0.07–3.63) |  |
|  | **b. Facility delivery cases only** | 0.06 | .12 |  | 0.13 | .25 |  | 0.04 | .06 |  | 0.06 | .09 |
|  | **(n = 2,157)** | (0.00–2.05) |  |  | (0.00–4.49) |  |  | (0.00–1.10) |  |  | (0.00–1.60) |  |
|  | **c. Non-facility delivery cases only** | 2.00 | .60 |  | 1.99 | .68 |  | 0.90 | .93 |  | 3.51 | .38 |
|  | **(n = 2,903)**** | (0.15–27.13) |  |  | (0.08–50.04) |  |  | (0.09–8.81) |  |  | (0.21-58.88) |  |
| **Quality Score 3:**  **Human and physical resources** | **a. All cases** | 0.44 | .20 |  | 1.77 | .45 |  | 0.38 | .08 |  | 1.06 | .93 |
|  | **(n = 5,060)** | (0.12–1.56) |  |  | (0.41–7.71) |  |  | (0.13–1.10) |  |  | (0.30–3.72) |  |
|  | **b. Facility delivery cases only** | 0.39 | .36 |  | 5.94 | .18 |  | 0.42 | .28 |  | 2.56 | .37 |
|  | **(n = 2,157)** | (0.05–2.85) |  |  | (0.43–81.95) |  |  | (0.08–2.05) |  |  | (0.33–19.68) |  |
|  | **c. Non-facility delivery cases only** | 0.71 | .68 |  | 0.90 | .90 |  | 0.56 | .44 |  | 0.56 | .51 |
|  | **(n = 2,903)**** | (0.14–3.63) |  |  | (0.16–5.11) |  |  | (0.13–2.40) |  |  | (0.10–3.17) |  |

OR = odds ratio, AOR = Adjusted odds ratio, 95% CI = 95% confidence interval.

*Adjusted for age, ethnicity, education, residential area, wealth index, intended pregnancy, multipara, involvement in decision-making on health, tobacco, and province.

**Omitted multiple pregnancies in perinatal deaths analysis. Other covariates are the same above.
