# Supplementary material for: Improving the quality of maternal and newborn healthcare at the district level: Addressing newborn deaths in Nepal
Source: PLOS Glob Public Health. 2023 Aug 2;3(8):e0002101. doi: 10.1371/journal.pgph.0002101 (PMC10395984; doi:10.1371/journal.pgph.0002101)
Supplement: Attachment — Submitted filename: Response to reviewers.docx [file pgph.0002101.s004.docx]

Author response to reviewers

*Dear Dr. Danish Ahmad and editorial team,*

*Thank you for giving us the opportunity to submit a revised draft of our manuscript titled “Improving the quality of maternal and newborn healthcare at the district level: addressing newborn deaths in Nepal” for publication in PLOS Global Public Health. We appreciate the time and effort that you and the reviewers dedicated to providing your valuable feedback on our manuscript. We are grateful to the reviewers for their insightful comments on our paper. We incorporated changes to reflect most of the suggestions provided by the reviewers. All line numbers refer to the revised manuscript file without tracked changes.*

*As a journal’s requirement, we added the checklist of “Inclusivity in global research” as a supporting information (Checklist S1).*

**Comments from the editor and reviewers**

1. Style consistency for numbers under 10 spell them out

***Authors’ response:*** *We modified the numbers in the main text as you suggested.*

2. Please use per cent instead of % in the text

***Authors’ response:*** *We modified “%” to “percent” in the main text, considering that the manuscript is written in the American English.*

3. 115-117 please qualify this line in the case of Nepal. The aspects mentioned in line 116 have been studied elsewhere with known care outcomes across the maternal-newborn spectrum.

***Authors’ response:*** *We added the sentences in Introduction as follows:*

“*According to a descriptive study assessed quality of primary care across 10 LMICs, composite and domain scores for quality of primary care facilities were low particular in user experience across 10 countries including Nepal. [8]” (lines: 96–98)*

4. Again in line 126 please qualify that the existent study gap is for Nepal. Care outcomes have been studied at the district level for South Asian countries.

***Authors’ response:*** *We deleted that sentence and added the sentences in Introduction as follows:*

*“Although many studies have focused on the coverage of routine maternal and newborn care and the availability of physical resources, few studies has systematically examined multidimensional maternal and newborn care quality, including experience of care [13,16,17]. No studies have examined district-level multidimensional care quality to date [18]. Although a study in another South Asian country examined the geographic variation of maternal and newborn care quality at the district level in another south Asian country, it also focused on inputs of routine care but not multidimensional care quality [19].” (lines: 117–123)*

5. Lines 135 Check intext referencing format and follow journal
   guidelines.Concurrent references are separated by a dash or comma, eg
   [21][22] should be written as [21,22].

***Authors’ response:*** *We modified the intext citation style in the main text as you suggested.*

6. Please explain in line 139 how the 992 health facilities were selected
   from the larger pool of health facilities. Do these include both
   public and private care facilities?

***Authors’ response:*** *Thank you for your suggestion. We added the sentences in Methods as follows:*

*“The selected facilities included all nonspecialized government hospitals, all private hospitals with 100 or more inpatient beds, and all primary health care centers. In addition, health posts, private hospitals whose beds between 15 and 99 beds, stand-alone HIV testing and counseling sites, and urban health centers were included a result of sample selection to set the total number of facilities as approximately 1,000.” (Lines:1464–149)*

7. Table 1: please list province names

***Authors’ response:*** *Thank you for your suggestion. NDHS 2016 data sets and final report used numbers to describe seven provinces and they did not use the specific name. So, we wrote provinces as “Province 1 to 7,” following their description.*

8. Please check table 2 formatting (NGO/private… shows % typos

***Authors’ response:*** *Thank you for your suggestion. We modified the number in that table.*

Review Comments to the Author
Reviewer #1: Although the analysis is rigorous and the data available in 2015 and 2016 have been used well for the purpose of the study, Newborn care particularly following newer studies related to skin-to-skin care and Kangaroo Mother Care now propose different approaches to Newborn care quality that were not known well during the surveys conducted in 2015 and 2016. Further later studies such as the SPA 2021 in Nepal provide richer information. It may be useful for the authors to note some of these advances and publications and how the quality of care is undergoing major shifts when it comes to newborn care and preterm babies. If not, the reader in 2023 may still use the parameters measured and surveyed in 2015 and 2016 for decisions
related to how one can make a major shift to reducing newborn mortality.

When it comes to Perinatal mortality issues of 3 delays related to complications occurring during pregnancy and how they were handled at the family level, at the time of transport from home to the facility and at the facility itself are important. Perhaps these can be included in the discussion even if there were no data. Maternal mortality and morbidity and the main causes of complications can occur regardless of the ANC quality and the authors could note these in their discussion.

*Thank you so much for your comments. We agree with your idea that the latest datasets will help assessing quality of care with richer quality parameters and will provide updated information. Owing to the nature of the data linkage, we chose the latest datasets whose survey year gap is close (NSPA 2015 and NDHS 2016). We would consider analyzing by linking NSPA 2021 and NDHS 2022 once the NDHS 2022 become publicly available as you suggested.*

*Thank you for your suggestion. We added the sentences in Discussions as follows:*

*“Also, the first and second delay in the three-delay model, which occurs before reaching health facilities, may have contributed more when it comes to the perinatal health outcome. Patient-oriented factors and transport from home to facilities may take longer time before labor started. [45].” (lines: 435–438)*

Reviewer #2: The topic is relevant to Nepal and in resource limited settings.
The narration was intelligently presented Analysis plan was detailed and Comprehensive
Results and discussion are in line with the research questions

*Thank you so much for your comments.*
