# Supplementary material for: Improving the quality of maternal and newborn healthcare at the district level: Addressing newborn deaths in Nepal
Source: PLOS Glob Public Health. 2023 Aug 2;3(8):e0002101. doi: 10.1371/journal.pgph.0002101 (PMC10395984; doi:10.1371/journal.pgph.0002101)
Supplement: Attachment — Submitted filename: Response to Reviewers.docx [file pgph.0002101.s005.docx]

Response to Reviewers

*Dear Dr. Danish Ahmad and editorial team,*

*Thank you for giving us the opportunity to submit a revised draft of our manuscript titled “Improving the quality of maternal and newborn healthcare at the district level: addressing newborn deaths in Nepal” for publication in PLOS Global Public Health. We are grateful to the reviewers for their feedback. We have incorporated changes to reflect the suggestions provided by the reviewers. Here is a point-by-point response to the reviewers’ comments. All line numbers refer to the revised manuscript file without tracked changes.*

Journal Requirements:

1. Please review your reference list to ensure that it is complete and correct. If you have cited papers that have been retracted, please include the rationale for doing so in the manuscript text, or remove these references and replace them with relevant current references. Any changes to the reference list should be mentioned in the rebuttal letter that accompanies your revised manuscript. If you need to cite a retracted article, indicate the article’s retracted status in the References list and also include a citation and full reference for the retraction notice.

*We checked the reference list and made correction on the bibliographic information of the referenced articles. We also checked that all references were not retracted. We did not add new articles in this round.*

2. Please insert an Ethics Statement at the beginning of your Methods section, under a subheading 'Ethics Statement'. It must include:

1) The name(s) of the Institutional Review Board(s) or Ethics Committee(s)

2) The approval number(s), or a statement that approval was granted by the named board(s)

3) (for human participants/donors) - A statement that formal consent was obtained (must state whether verbal/written) OR the reason consent was not obtained (e.g. anonymity). NOTE: If child participants, the statement must declare that formal consent was obtained from the parent/guardian.

*We modified the subheading and moved the contents in relation to ethics statement to the beginning of the Methods section. (Lines:136–140)*

Additional Editor Comments (if provided):

Thank you for revising the paper-there are two un addressed comments that the authors should address
Please correct the typo in these lines-"Although a study in another South Asian country 123 examined the geographic variation of maternal and newborn care quality at the district level in 124 another south Asian country, it also focused on inputs of routine care but not multidimensional 125 care quality [19].

*Thank you for your comment. We modified the sentence as follows:*

*“Although a study in another South Asian country examined the geographic variation of maternal and newborn care quality at the district level, it also focused on inputs of routine care but not multidimensional care quality [19].” (Lines: 120­–123)*

2. referring to comment 7 where province names were requested to be listed, the NDHS 2016 report on page 13 provides a map with corresponding province numbers-can you pleae try to add provinvce names by reviewing the map and matching against exisitn names. Publishing a paper with province numbers and not numbers limitts interpretation for international and importantly researchers/policy and program managers in Nepal

*Thank you for your comment. We added the province names in the tables (Table1 in Main text and Table S2 in Additional file).*
